# Antibiogram, antibiotic resistance genes and health risks of *Listeria *species from fruits and vegetables in selected locations in Nigeria

**DOI:** 10.12688/f1000research.171697.1

**Published:** 2025-12-22

**Authors:** Anthony A. Adegoke, Kate O Igoche, Olaniyi Owolawi, Moses Ekpenyong, Joseph Omololu-Aso, Olayinka A Aiyegoro

**Affiliations:** 1 Department of Microbiology, University of Uyo, Uyo, Akwa Ibom, Nigeria; 2 Faculty of Health Sciences, Durban University of Technology, Durban, KwaZulu-Natal, South Africa; 3 Department of Science Education, Faculty of Education, University of Nigeria, Nsukka, Enugu State, Nigeria; 4 Department of Computer Science, Faculty of Computing, University of Uyo, Uyo, Nigeria; 5 Department of Microbiology, Obafemi Awolowo University Faculty of Science, Ile-Ife, Nigeria; 6 Research Unit for Environmental Sciences and Management, North-West University Private Bag, X1290 Potchefstroom 2520, South Africa

**Keywords:** Listeria monocytogenes, antimicrobial resistance, Quantitative Microbial Risk Assessment (QMRA), fresh produce, Food safety

## Abstract

**Background:**

Fresh produce is a vital vehicle for the transmission of foodborne pathogens, yet data from low- and middle-income countries on contamination levels and associated health risks remain scarce.

**Methods:**

This study provided an analysis of *Listeria* species contamination in 208 fruit and vegetable samples from major geopolitical regions in Nigeria. Conventional microbiological techniques and molecular methods were used for the analysis.

**Results:**

We reported a high prevalence (38.5%) and substantial bacterial loads (up to 8.33×10 ^2^ CFU/25g) among the organically grown crops, with produce from Northern states exhibiting the highest contamination. Genetic characterisation revealed significant diversity, including novel strains and others closely related to global clones, confirming a resilient environmental presence. Importantly, antibiotic susceptibility testing uncovered alarming resistance rates to vancomycin (83.1%) and oxacillin (88.1%), with hierarchical clustering identifying distinct multidrug-resistant clusters. The presence of resistance genes ( *vanA, vanB, tetL, tetM*) and the virulence gene *hly* was confirmed. A quantitative microbial risk assessment (QMRA), incorporating this empirical data, estimated the annual probability of illness, highlighting high-risk produce-region combinations.

**Conclusions:**

These findings demonstrate that fresh produce in Nigeria is a significant reservoir of pathogenic, antimicrobial-resistant *L. monocytogenes*, posing a serious public health threat. This study highlights the urgent need for targeted interventions, including enhanced agricultural water safety and strengthened surveillance of antimicrobial resistance, to mitigate risks and support evidence-based food safety policies in Nigeria and similar regions.

## 1. Introduction

The global food system is increasingly reliant on the production and distribution of fresh fruits and vegetables, which are fundamental components of a healthy diet due to their rich nutritional composition. ^1^ However, the consumption of raw or minimally processed fresh produce is a well-documented route for the transmission of foodborne pathogens, posing a significant public health risk worldwide. ^2^ Among these pathogens, *Listeria monocytogenes*, a facultative anaerobic, Gram-positive bacterium, is of particular concern due to its severe clinical manifestations and high mortality rates of 20-30% in susceptible populations. ^3^ Listeriosis primarily affects immunocompromised individuals, pregnant women, newborns, and the elderly, often leading to septicemia, meningitis, and spontaneous abortions. ^4^ A critical characteristic of *L. monocytogenes* is its psychrotrophic nature, enabling it to survive and multiply at refrigeration temperatures, which challenges conventional food preservation strategies and extends its shelf-life within the food chain. ^5^

In low- and middle-income countries (LMICs), the risk of produce contamination is exacerbated by factors such as the widespread use of untreated wastewater or polluted water sources for irrigation, inadequate sanitation facilities, and the challenges of maintaining cold chains during storage and transport. ^6– 8^ Nigeria, as a populous nation in Sub-Saharan Africa with a rapidly growing urban population dependent on market-oriented agriculture, is particularly vulnerable to such challenges. Several studies have reported the occurrence of *Listeria* species and other enteric pathogens on fresh produce within the country, ^9, 10^ highlighting a potential, yet under-quantified, public health threat.

Compounding this biological hazard is the escalating global crisis of antimicrobial resistance (AMR), which threatens the effective treatment of bacterial infections. The imprudent use of antibiotics in human medicine, veterinary practice, and agriculture has selected for resistant bacteria, including *L. monocytogenes*, thereby diminishing therapeutic options and increasing the severity of listeriosis outcomes. ^11^ The transfer of antibiotic resistance genes (ARGs) among bacteria in environmental niches, such as soil and water used in agriculture, further facilitates the dissemination of resistance mechanisms. ^12^ While surveillance data on AMR in foodborne pathogens is comprehensive in many high-income countries, significant gaps remain in the data from LMICs, including Nigeria. ^13^

Quantitative Microbial Risk Assessment (QMRA) is a structured, science-based framework recommended by the Codex Alimentarius Commission for estimating the risk of illness from exposure to pathogenic microorganisms in food. ^14^ By integrating data on pathogen prevalence, concentration, consumption patterns, and dose-response relationships, QMRA models provide a powerful tool for identifying high-risk food-pathogen combinations, informing risk management decisions, and prioritising public health interventions. ^15^ The application of QMRA to *L. monocytogenes* in ready-to-eat foods is well-established in developed nations; however, its application in the context of African food systems and traditional produce is scarce.

Therefore, this study aims to bridge these critical knowledge gaps by providing a comprehensive analysis of *Listeria monocytogenes* contamination in organically grown fresh fruits and vegetables from major geopolitical regions of Nigeria. We quantify the microbial load, molecularly characterise the isolates to determine diversity and relatedness to global strains, and profile their phenotypic and genotypic resistance to important antibiotics. Crucially, we integrate this empirical data into a robust QMRA model to estimate the annual risk of illness from consumption of contaminated produce. This holistic approach—from farm to fork to public health outcome—will yield vital data for evidence-based food safety policies, targeted surveillance programs, and antimicrobial stewardship initiatives in Nigeria and other regions with similar socio-agricultural landscapes.

## 2. Materials and methods

### 2.1 Study areas and sample collection

The study was conducted across six major geopolitical regions in Nigeria: South-South (Akwa-Ibom), South-West (Ile-Ife), North (Plateau, Kano and Bauchi), and South-East (Enugu). Further analysis in Plateau state was dropped due to insecurity as further sampling could not be done. Within each region, sampling points were strategically selected to encompass major farms known to utilise animal waste manure for fertilisation and wastewater or polluted water sources for irrigation purposes. About 30 samples were collected from farms not using such manure, because there were cow droppings within and around the farmland (inadvertent manuring), which obviously were expected to have impacts on the crops.

A total of 208 fruit and vegetable samples were aseptically collected. Samples included Water Leaf, Cabbage, Carrot, Pumpkin, Lady Finger, Lettuce, Amaranth, Ugwu, Ora, Onugbu, Nchuanwu, Garden Egg, and Water Melon. For each produce type at a given location, a composite sample was created by pooling sub-samples from at least five different sampling points (e.g., five different vendors or farm plots) to obtain a representative average for that location. Each composite sample was placed in a sterile stomacher bag, stored in a cool box at 4°C, and transported to the laboratory for analysis within 6 hours of collection.

### 2.2 Listerial quantification and isolation procedure

Quantification of *Listeria* spp. was performed using a standard plate count technique following a ten-fold serial dilution. Briefly, 25 g of each composite sample was homogenised in 225 mL of Buffered Peptone Water (BPW) for 2 minutes using a stomacher. The homogenate was then serially diluted from 10 ^−1^ to 10 ^−6^ in 0.1% peptone water.

From appropriate dilutions, 1 ml aliquots were spread-plated in duplicate onto two selective agars: Oxford Listeria Selective Agar (Oxoid) and Microinstant ^®^ Listeria Selective Agar (Scharlab). All plates were incubated aerobically at 37°C for 24-48 hours. For Southwest analysis, pre-enriched incubation in serially diluted Half-Fraser Broth and Full-Fraser Broth (OXOID, CM0895 ISO, United Kingdom) at 37 ^o^C for 24-48 h. Characteristic colonies on both media were counted, and the results were expressed as Colony Forming Units per 25 grams of sample (CFU/25g).

The mean count for each produce-type and region combination was calculated from the duplicate plate counts of the composite sample. This mean value, derived from the two-culture media, was used as the definitive concentration (Mean CFU/25g) for all subsequent risk assessment calculations.

### 2.3 Purification and subculture of isolates for characterisation

Selected bacterial colonies were purified through subculturing onto fresh agar plates to obtain axenic cultures essential for subsequent phenotypic and genotypic characterisation. ^16^

### 2.4 Phenotypic identification of *Listeria* spp.

Presumptive *Listeria* isolates were subjected to a series of biochemical assays for preliminary identification. Initial characterisation included Gram-staining, which revealed Gram-positive, rod-shaped morphology. This was followed by a catalase test, which yielded a positive result, and an oxidase test, which produced a negative reaction. Haemolytic reaction in 5% sheep red blood agar was examined with *Listeria monocytogenes* and *L. ivanovii* expected to show β haemolysis, while *L. innocua* is expected to show γ haemolysis. This specific combination of results is consistent with the typical biochemical profile of the genus *Listeria.* ^16^

### 2.5 Molecular identification of presumptive *Listeria* isolates

**2.5.1 DNA extraction, visualisation, and quantification**

Genomic DNA was isolated from the purified bacterial cultures using the ZR Fungal/Bacterial DNA Miniprep kit (Zymo Research, USA), following the manufacturer’s instructions. To confirm the success of the extraction and assess DNA integrity, the eluate was analysed by agarose gel electrophoresis. A 1% agarose gel, prepared with Tris/Borate/EDTA (TBE) buffer and stained with ethidium bromide, was loaded with the DNA samples alongside a molecular weight marker. Electrophoresis was performed at 100 volts for one hour, after which the DNA fragments were visualised under ultraviolet light using a UV transilluminator (BIORAD, South Africa). The concentration and purity (A260/A280 ratio) of the extracted DNA were subsequently determined using a NanoDrop 2000 spectrophotometer (Thermo Scientific, UK). ^17^

**2.5.2 Amplification and sequencing of genetic materials**

The 16S rRNA gene was targeted for amplification to enable precise species identification. Each 25 μL polymerase chain reaction (PCR) mixture contained 12.5 μL of Taq 2X Master Mix (New England Biolabs, USA), 1 μL of each 10 μM forward (27F: 5'-AGAGTTTGATCMTGGCTCAG-3') and reverse (1525R: 5'-AAGGAGGTGWTCCARCCGCA-3') primers, 2 μL of DNA template, and 8.5 μL of molecular grade water. The thermocycling conditions comprised an initial denaturation at 94°C for 5 minutes; 36 cycles of denaturation (94°C for 30 seconds), annealing (55°C for 30 seconds), and extension (72°C for 45 seconds); followed by a final extension at 72°C for 7 minutes before holding at 10°C.

**2.5.3 Visualisation of amplified fragments**

To confirm successful amplification of the target gene, the PCR products were electrophoresed on a 1% agarose gel as described in the previous section, using a DNA ladder to estimate the amplicon size.

**2.5.4 Gene sequencing and data analysis**

Amplified PCR products were purified in preparation for sequencing. A sequencing reaction mixture was prepared by combining 1 μL of purified PCR product with 9 μL of Hi-Di Formamide. The samples were then sequenced bidirectionally using the Sanger method on a 3130xl Genetic Analyser (Applied Biosystems, USA).

**2.5.5 Sequence submission to GenBank**

The obtained 16S rRNA gene sequences were deposited into the GenBank database to obtain accession numbers, ensuring the data is publicly available for future research. ^18^

**2.5.6 Sequence alignments and phylogenetic investigation**

The resulting forward and reverse sequence reads were assembled and edited to generate a consensus sequence using BioEdit software (version 7.0.5.3). This consensus sequence was used to query the GenBank database via the BLASTN algorithm on the National Centre for Biotechnology Information (NCBI) platform to identify the closest known species matches. For phylogenetic inference, related sequences were downloaded and aligned using ClustalW. A phylogenetic tree was constructed using the Maximum Likelihood method based on the Tamura-Nei model in MEGA 7 software (version 7.0.63). ^19^

### 2.6 Quantitative Microbial Risk Assessment (QMRA) Methodology

A quantitative microbial risk assessment (QMRA) was conducted to estimate the probability of illness from *Listeria monocytogenes* associated with the consumption of contaminated fresh produce. The assessment followed the internationally recognised four-step framework: hazard identification, exposure assessment, dose-response assessment, and risk characterisation. ^14, 20^

**2.6.1 Hazard identification**

The pathogen of concern was identified as *Listeria monocytogenes*, a facultative anaerobic, Gram-positive bacterium known to cause the severe foodborne illness listeriosis. The hazard was characterised by its ability to grow at refrigeration temperatures and its high mortality rate, particularly among immunocompromised individuals, pregnant women, newborns, and the elderly.

**2.6.2 Exposure assessment**

The exposure assessment aimed to estimate the number of *L. monocytogenes* cells ingested per serving of a given produce type. The initial concentration of *Listeria* spp. C_listeria_spp, measured in colony-forming units per 25 grams (CFU/25 g) as detailed in Section 3.4, served as the starting point. This value was converted to a concentration per gram and adjusted to reflect the concentration of the pathogenic species, *L. monocytogenes* (C_path), using Equation 1.

C_path(CFU/g)=[(Mean_CFU/25g)/25]×F_path
Equation 1

Where:

✓ Mean CFU/25g is the mean count derived from the two-enumeration media (Oxford and Microinstant ^®^ Listeria) for each produce-region combination.
✓ F_path is the fraction of *Listeria* spp. presumed to be *L. monocytogenes.* A conservative value of 0.10 (10%) was used based on the reported variability in the prevalence of the pathogenic species within the genus. ^21, 22^

The daily exposure dose (D) was then calculated using Equation 2.

D(CFU/serving)=C_path×R×P×S
Equation 2

Where:

✓ R is the reduction factor accounting for preparation steps (e.g., washing, cooking). A value of 1 was applied, representing a worst-case scenario where no reduction occurs before consumption.
✓ P is the prevalence of contamination. A value of 1 (100%) was assumed, indicating that every serving is contaminated, providing a conservative estimate of risk.
✓ S is the serving size in grams. A standard serving size of 100 g was used for all produce types.

2.6.3 Dose-response assessment

The probability of infection per serving was estimated using the exponential dose-response model ( Equation 3), which is commonly applied for pathogenic bacteria including *L. monocytogenes.*

P_infection=1−exp(−r×D)
Equation 3

Where:

✓ P_infection is the probability of infection per eating occasion.
✓ r is the host-pathogen infectivity parameter, representing the probability that a single viable cell will initiate an infection. A value of r = 1.18 × 10 ^−12^ was used, as established in previous risk assessments for *L. monocytogenes.* ^23– 25^

The probability of illness (P_ill) was subsequently derived by accounting for the proportion of infections that lead to clinical symptoms, as shown in Equation 4.

P_ill=P_infection×P_ill|inf
Equation 4

Where:

✓ P_ill|inf is the conditional probability of illness given that infection has occurred. A value of 0.5 (50%) was selected to represent a highly susceptible population subset. ^26^

**2.6.4 Risk characterization**

The final annual risk of illness was calculated by integrating the per-serving risk with the frequency of consumption throughout the year. The probability of illness after f exposures was calculated using the exponential annual risk model ( Equation 5).

$$P\_\mathrm{ill}\_\text{annual}=1-{\left(1-P\_\mathrm{ill}\right)}^{f}$$
Equation 5

Where:

✓ f is the annual consumption frequency, assumed to be 12 times per year (once per month) for each produce type.

For the very low values of P_ill calculated in this assessment, Equation 5 can be approximated by P_ill_annual ≈ P_ill × f.

### 2.7 Antibiotic susceptibility profiling

The antibiotic susceptibility profile of the selected Listeria species isolates was determined using the standardised disk diffusion technique (Kirby-Bauer method) as outlined by the Clinical and Laboratory Standards Institute (CLSI, 2020). A panel of seven antibiotics was selected to represent major classes of antimicrobial agents. These included amoxicillin-clavulanic acid (AUG), cefoxitin (CFX), ceftriaxone (CRO), ofloxacin (OFX), imipenem (IMI 10), vancomycin (Va 30), and oxacillin (Ox 5). Before testing, each bacterial isolate was revived through two successive subcultures on Brain Heart Infusion agar at 37°C for 24 hours to ensure purity and metabolic activity. A standardised bacterial inoculum was prepared by suspending several well-isolated colonies in sterile saline to achieve a turbidity equivalent to 0. 0.5 McFarland standard, approximately 1–2 × 10 ^8^ CFU/mL.

This standardised suspension was then evenly swabbed onto the surface of Mueller-Hinton Agar plates. Antibiotic discs of specified potencies were aseptically placed onto the inoculated agar surface, ensuring sufficient spacing to prevent overlapping zones of inhibition. The plates were incubated at 35°C ± 2°C for 16–18 hours. Following incubation, the diameters of the zones of complete inhibition were measured to the nearest millimetre using a calibrated digital calliper. The results were interpreted based on the clinical breakpoints provided by CLSI (2020). It is important to note that, as specific interpretive criteria for Listeria species are not available for all antibiotics, the breakpoints for cefoxitin and oxacillin were applied based on standards for *Staphylococcus* spp., a common practice in studies of Listeria susceptibility. For clear and conservative data analysis in this study, any result interpreted as ‘Intermediate’ was categorised as ‘Resistant’ to provide a more stringent assessment of non-susceptibility.

The resulting susceptibility data were analysed and visualised using the Python programming language, version 3.12.11. The overall resistance prevalence was depicted using a simplified stacked bar chart, which clearly illustrated the percentage of isolates classified as resistant or sensitive for each antibiotic, providing an immediate macroscopic view of resistance trends. To explore the patterns of multidrug resistance and relationships between the isolates, a hierarchical clustering heatmap was constructed. This analysis employed the Jaccard distance metric and average linkage method to cluster both the bacterial isolates based on their antibiograms and the antibiotics based on their co-resistance patterns. This dual dendrogram heatmap effectively identified clusters of multidrug-resistant strains and groups of antibiotics to which resistance was commonly linked, offering a sophisticated visual representation of the complex susceptibility profile data.

### 2.8 Detection of antibiotic and virulence genes

The detection of antimicrobial resistance genes ( *vanA, vanB, tetL,* and *tetM*) and the virulence gene *hly* (encoding listeriolysin O) in the identified *Listeria* species was performed using multiplex polymerase chain reaction (PCR) assays. The protocol for amplifying the resistance genes was adapted from the multiplex PCR method established by Iweriebor *et al*., ^27^ utilising the primer sequences and thermocycling conditions detailed in Table 1. Similarly, the detection of the *hly* virulence gene was carried out via a uniplex PCR assay following the methodology described by Kumar *et al.,* ^28^ which is a well-characterised and specific protocol for identifying this critical virulence factor in *Listeria monocytogenes.*

**Table 1.** List of primers for the detection of resistance genes and virulence genes.

	Gene	Primer sequence (5'-3')	Product size (bp)
Antibiotic resistance genes	*vanA*	F-GCGCGGTCCACTTGTAGATA	314
		R-TGAGCAACCCCCAAACAGTA	
	*vanB*	F-AGACATTCCGGTCGAGGAAC	220
		R-GCTGTCAATTAGTGCGGGAA	
	*tetK*	F-TCGATAGGAACAGCAGTA	169
		R-CAGCAGATCCTACTCCTT	
	*tetL*	F-TCGTTAGCGTGCTGTCATTC	267
		R-GTATCCCACCAATGTAGCCG	
	*tetM*	F-GTATCCCACCAATGTAGCCG	406
		R-CGGTAAAGTTCGTCACACAC	
Virulence gene	*hlyA*	F-ATTTTCCCTTCACTGATTGC	276
		R-CACTCAGCATTGATTTGCCA	

The polymerase chain reaction (PCR) was performed in a final volume of 25 μL. This reaction mixture contained 12.5 μL of a commercial master mix (OneTag Quick Load, New England BioLabs), 5 μL of DNA template, and primers at concentrations ranging from 0.5 μM to 1.25 μM, with the remaining volume adjusted using nuclease-free water. The thermocycling protocol began with an initial denaturation and enzyme activation step at 94°C for five minutes. This was followed by 35 amplification cycles, each involving denaturation at 94°C for 60 seconds, primer annealing at 55°C for 60 seconds, and strand extension at 72°C for 90 seconds. To ensure complete elongation of any nascent DNA strands, a final extension was carried out at 72°C for 15 minutes. The PCR products were then visualized by resolving them on a 2% agarose gel via electrophoresis at 110 V for 45 minutes. Amplicon sizes were determined by comparison to a 100 bp DNA ladder.

PCR was conducted at 60°C with a gradient of 2°C, utilizing Taq polymerase at a concentration of 0.5–3.0 units, MgCl _2_ at 1–3.0 mM, and primers at 25–100 ng. The annealing duration was 60 seconds, while the extension times were set at 30 seconds and 1 minute. The total number of cycles performed was 40, employing four sets of primers in the multiplex PCR assay.

## 3. Results

### 3.1 Quantification and isolation, occurrence and frequencies of occurrence of *Listeria* species

The results of the microbiological analysis, which quantified *Listeria* species contamination across various fresh produce types from different geopolitical regions in Nigeria, are summarised in Table 2. The table presents the average bacterial counts, expressed in colony-forming units per 25 grams (CFU/25 g), obtained from two selective culture media: Oxford Listeria (Oxoid) and Microinstant ^®^ *Listeria* (Scharlab). The final composite average count for each produce-type and region combination was calculated from these values. A total of 208 samples were analysed, of which 80 were positive for *Listeria* species, resulting in an overall occurrence rate of 38.5%. The data reveal substantial variation in contamination levels and frequency, with produce from Northern regions such as Kano and Bauchi generally exhibiting the highest bacterial loads, notably in samples of Lady finger (8.33 × 10 ^2^ CFU/25 g) and Amaranth (7.43 × 10 ^2^ CFU/25 g). In contrast, produce including Pumpkin and Ora from various regions showed the lowest levels of contamination.

**Table 2.** Enumeration of *Listeria* species Fruit and Vegetable Samples.

Produce Type	Region	No of Samples	No Positive (Occurrence)	% Positive (Frequencies of Occurrence)	Average Count per Enumeration media (CFU/25 g)		Average Count (CFU/25 g)
					Oxford (Oxoid)	Microinstant ^®^ Listeria (Scharlab)	
Water Leaf	South South (Akwa-Ibom)	21	9	42.9	3.25 × 10 ^2^	2.60 × 10 ^2^	2.93 × 10 ^2^
	South West (Ile-Ife)	18	5	27.8	2.50 × 10	5.00 × 10 ^1^	3.75 × 10 ^1^
Cabbage (kabeji)	North (Kano)	9	3	27.3	6.75 × 10 ^2^	5.40 × 10 ^2^	6.08 × 10 ^2^
Carrot	North (Bauchi)	30	3	42.9	5.70 × 10 ^2^	4.50 × 10 ^2^	5.13 × 10 ^2^
Pumpkin	North (Kano/Bauchi)	11	4	36.4	2.50 × 10	2.00 × 10 ^1^	2.25 × 10 ^1^
Lady finger (Mata yatsa)	North (Kano/Bauchi)	7	4	57.1	9.25 × 10 ^2^	7.40 × 10 ^2^	8.33 × 10 ^2^
Lettuce (lecttuse)	North (Kano/Bauchi)	11	5	45.5	5.50 × 10 ^2^	5.50 × 10 ^2^	4.95 × 10 ^2^
Amaranth	North (Kano)	9	4	44.4	8.25 × 10 ^2^	6.60 × 10 ^2^	7.43 × 10 ^2^
Ugwu	South East (Enugu)	11	5	45.5	2.42 × 10 ^2^	1.94 × 10 ^2^	2.18 × 10 ^2^
Ora	South East (Enugu)	9	4	44.4	2.5 × 10	2.00 × 10 ^1^	2.25 × 10 ^1^
Onugbu	South East (Enugu)	10	6	60.0	2.25 × 10 ^2^	1.80 × 10 ^2^	2.03 × 10 ^2^
Nchuanwu	South East (Enugu)	9	3	33.3	1.75 × 10 ^2^	1.40 × 10 ^2^	1.58 × 10 ^2^
Garden egg	South West (Ile-Ife)	30	11	36.7	2.1 × x 10 ^2^	175 × 10 ^2^	1.89 × 10 ^2^
Water Melon	South South (Akwa-Ibom)	15	10	66.7	2.5 × 10	3.00 × 10 ^2^	1.13 × 10 ^2^
	South West (Ile-Ife)	8	3	37.5	1.25 × 10 ^2^	1.00 × 10 ^2^	1.13 × 10 ^2^
				208			

### 3.2 Isolated *Listeria* identity

Table 3 (a) and (b) present the identities and accession numbers of *Listeria* strains characterised in this study, with emphasis on their genetic distinctiveness and relatedness to previously reported strains. Table 3(a) shows the de novo sequenced *Listeria monocytogenes* and *Listeria innocua* isolates that were successfully deposited in the NCBI database, thereby confirming their novelty and genomic uniqueness. In contrast, Table 3(b) highlights *Listeria monocytogenes* isolates with varying percentages of identity to established reference strains, reflecting the degree of genomic similarity or divergence among the isolates. Collectively, these results demonstrate both the presence of unique local strains and the close genetic relationship of others to globally reported *Listeria* strains, underscoring the diversity within the isolates recovered in this study.

**Table 3.** (a) and (b): Bacterial identities, sources and accession number.

(a) Unique Identity of *Listeria* strains ( *De-novo *) as deposited and approved by NCBI, USA	
Identity	Accession number
*Listeria monocytogenes* strain UYO17	PX282429.1
*Listeria monocytogenes* strain UYY_IK21	PX282430.1
*Listeria monocytogenes* strain UYO21AB	PX282431.1
*Listeria monocytogenes* strain UYO23	PX282432.1
*Listeria monocytogenes* strain UYO30B	PX282433.1
*Listeria monocytogenes* strain UYO33A	PX282434.1
*Listeria monocytogenes* strain UYO-14AF2	PX282435.1
*Listeria monocytogenes* strain UYY47C	PX282436.1
*Listeria monocytogenes* strain UYY53	PX282437.1
*Listeria monocytogenes* strain UYYAF_07D	PX282438.1
*Listeria innocua* strain UYY_AK19A	PX282439.1
*Listeria monocytogenes* strain UYY_AF_63	PX282440.1
*Listeria monocytogenes* strain UYY_AF_63	PX282441.1
*Listeria monocytogenes* strain UYY_71C	PX282442.1
*Listeria monocytogenes* strain IK_X47A	PX282443.1
*Listeria monocytogenes* strain SW_IFE_001E	PX282444.1
*Listeria monocytogenes* strain SW_IFE_007A	PX282445.1
*Listeria monocytogenes* strain SE_NSUK_011C	PX282446.1
*Listeria monocytogenes* strain SW_IFE_005D	PX282447.1
*Listeria monocytogenes* strain SW_IFE_28A	PX282448.1
*Listeria monocytogenes* strain SW_IFE_22C	PX282449.1
*Listeria monocytogenes* strain SE_NSUK_014A	PX282450.1
*Listeria* monocytogenes strain NG_NE_111	PX282451.1
*Listeria* monocytogenes strain NW_KN_013	PX282452.1
*Listeria monocytogenes* strain NW_KN_96	PX282453.1

b. Identities of other *Listeria* strains and their percentage of identity to other references		
Identity	% Identity	Accession number
*Listeria monocytogenes* isolate UYO28AF	88.89	CP062127.1
*Listeria monocytogenes* isolate UYY-AF22C	96.20	MZ675510.1
*Listeria monocytogenes* isolate UYY_IK_17	93.01	MZ675515.1
*Listeria monocytogenes* isolate IK_12C	90.40	OK085526.1
*Listeria monocytogenes* isolate UYY_83	94.42	CP111150.1
*Listeria monocytogenes* isolate UYY66D	94.86	MT990525.1
*Listeria monocytogenes* isolate UYY_AF_82E	93.01	MT990565.1
*Listeria monocytogenes* isolate UYO_W45	93.61	OK642584.1
*Listeria monocytogenes* isolate UYY_AF_13E	89.55	CP168884.1
*Listeria monocytogenes* isolate NG_NW_17B	99.15	CP019619.1

### 3.3 Phylogenetic tree

**Figure 1.**
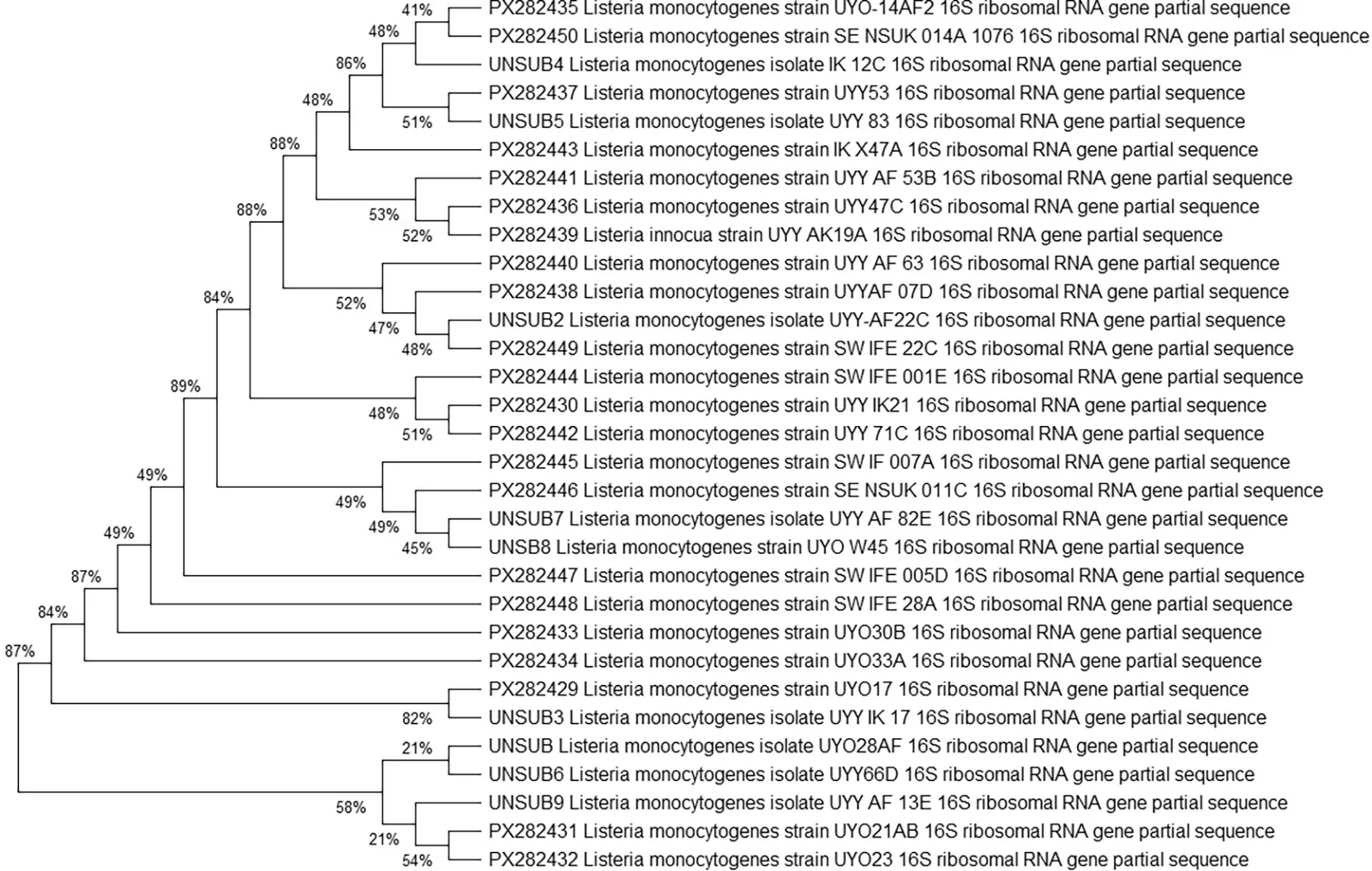
Phylogenetic relationships of the *Listeria* species from selected Nigerian locations.

To elucidate the genetic relatedness and evolutionary relationships among the *Listeria* isolates collected in this study, a phylogenetic tree was constructed based on partial 16S rRNA gene sequences. The analysis included a total of thirty-two isolates recovered from various locations and sources across Nigeria. The sequences were aligned, and a phylogenetic tree was inferred using the Maximum Likelihood method. As shown in Figure 1, the isolates clustered into distinct clades, confirming their identification as *Listeria monocytogenes* and revealing their phylogenetic grouping. Notably, the tree also illustrates the genetic diversity and potential evolutionary paths among the strains, with bootstrap values indicating the robustness of the major nodes. The inclusion of a *Listeria innocua* strain (PX282439) served as an outgroup to root the tree and provide a point of evolutionary reference.

### 3.4 Antibiogram of the *Listeria* species

**Figure 2.**
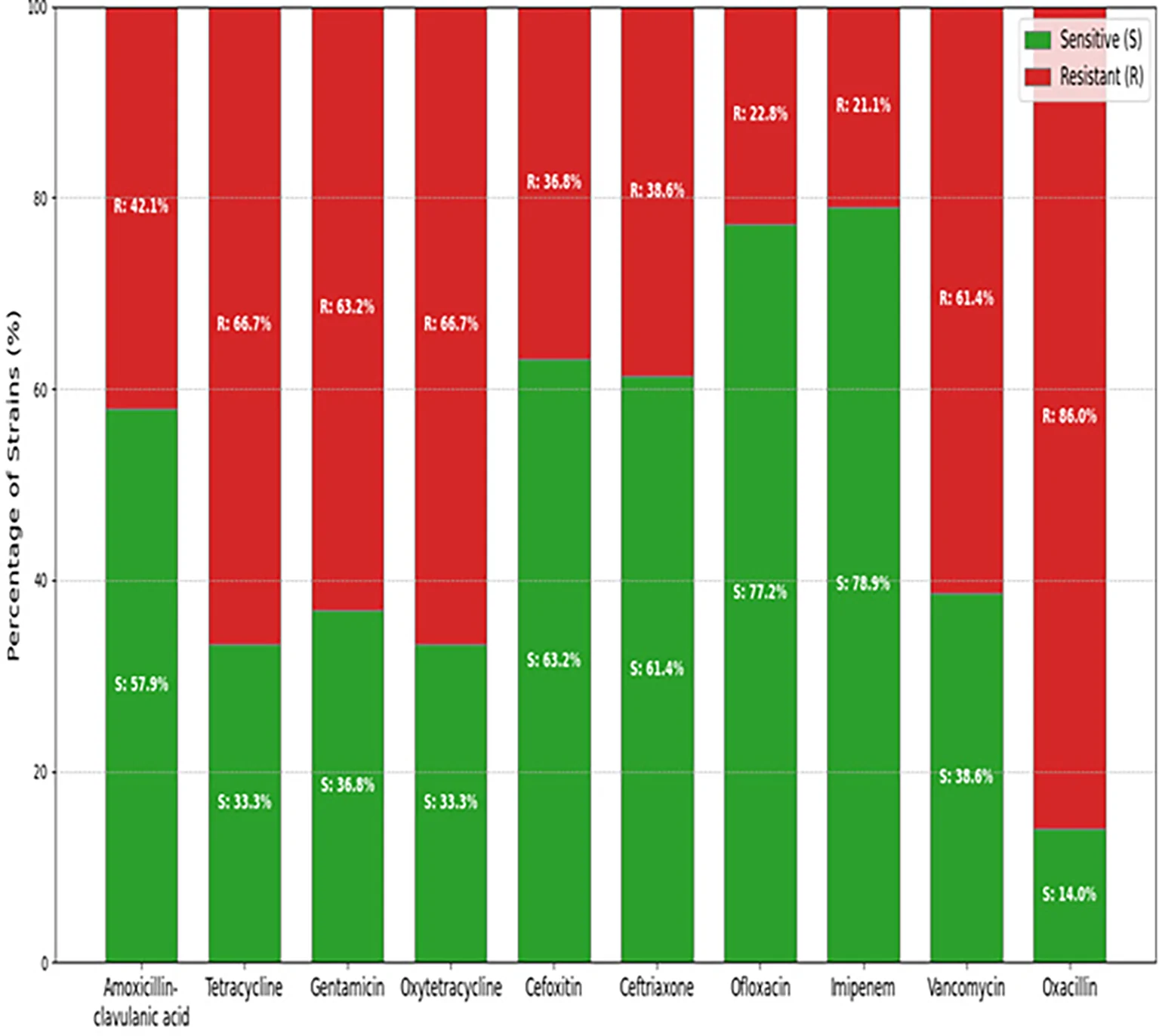
Antibiogram of selected *Listeria* species.

Figure 2 depicts the antibiotic resistance profiles of 156 Listeria strains across seven different antibiotics. The stacked bar chart shows the percentage of strains classified as sensitive (S) and resistant (R) for each antibiotic. Notably, Vancomycin and Oxacillin exhibit high percentages of resistant strains (83.1% and 88.1% respectively), while Ofloxacin has the highest percentage of sensitive strains (81.4%). This figure offers a clear visual summary of the resistance patterns observed in the tested *Listeria* isolates. While Figure presents the antibiotic profile, other subsequent figures present the pattern and relationships with other factors

### 3.5 Hierarchical clustering heatmap of scaled antibiotic resistance and virulence profiles across the *Listeria* strains

**Figure 3.**
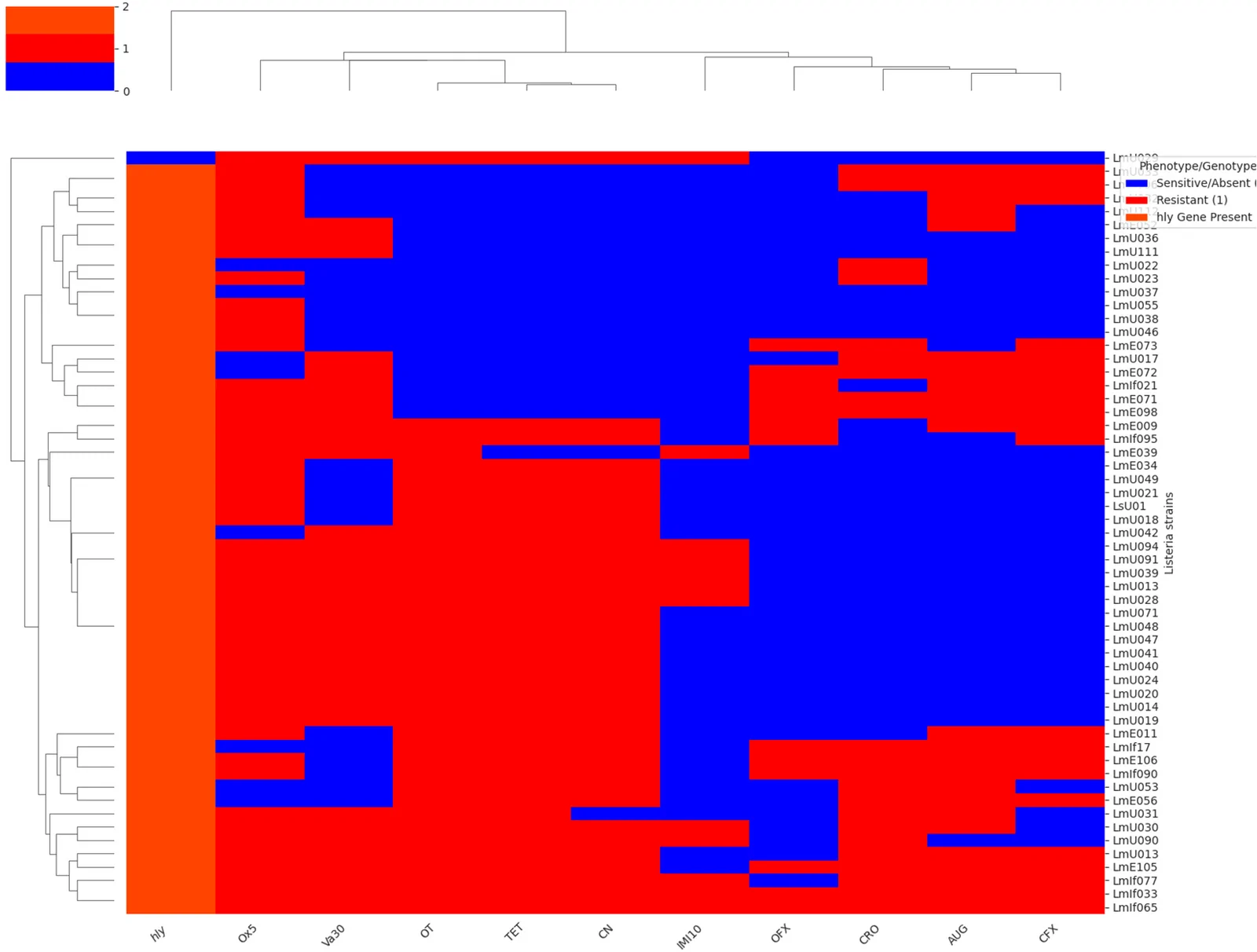
Hierarchical clustering heatmap of scaled antibiotic resistance profiles and *hly* genes across selected *Listeria* strains.

Figure 3 displays a hierarchical clustering heatmap that visualises the antibiotic resistance profiles and the presence of specific resistance genes across the *Listeria* strains analysed. The heatmap employs a colour gradient where blue represents sensitive or gene absence, red indicates resistance, and reddish-orange signifies the presence of a resistance gene. The dendrograms flanking the heatmap illustrate the hierarchical clustering of both the *Listeria* strains (rows) and the antibiotic/gene markers (columns), highlighting groups with similar patterns. The x-axis is labelled with the antibiotic names and italicised resistance gene names, while the y-axis shows the individual *Listeria* strains. This figure facilitates the identification of correlations between observed antibiotic resistance phenotypes and the presence of the investigated resistance genes, revealing potential relationships and clustering patterns within the *Listeria* isolates.

**Figure 4.**
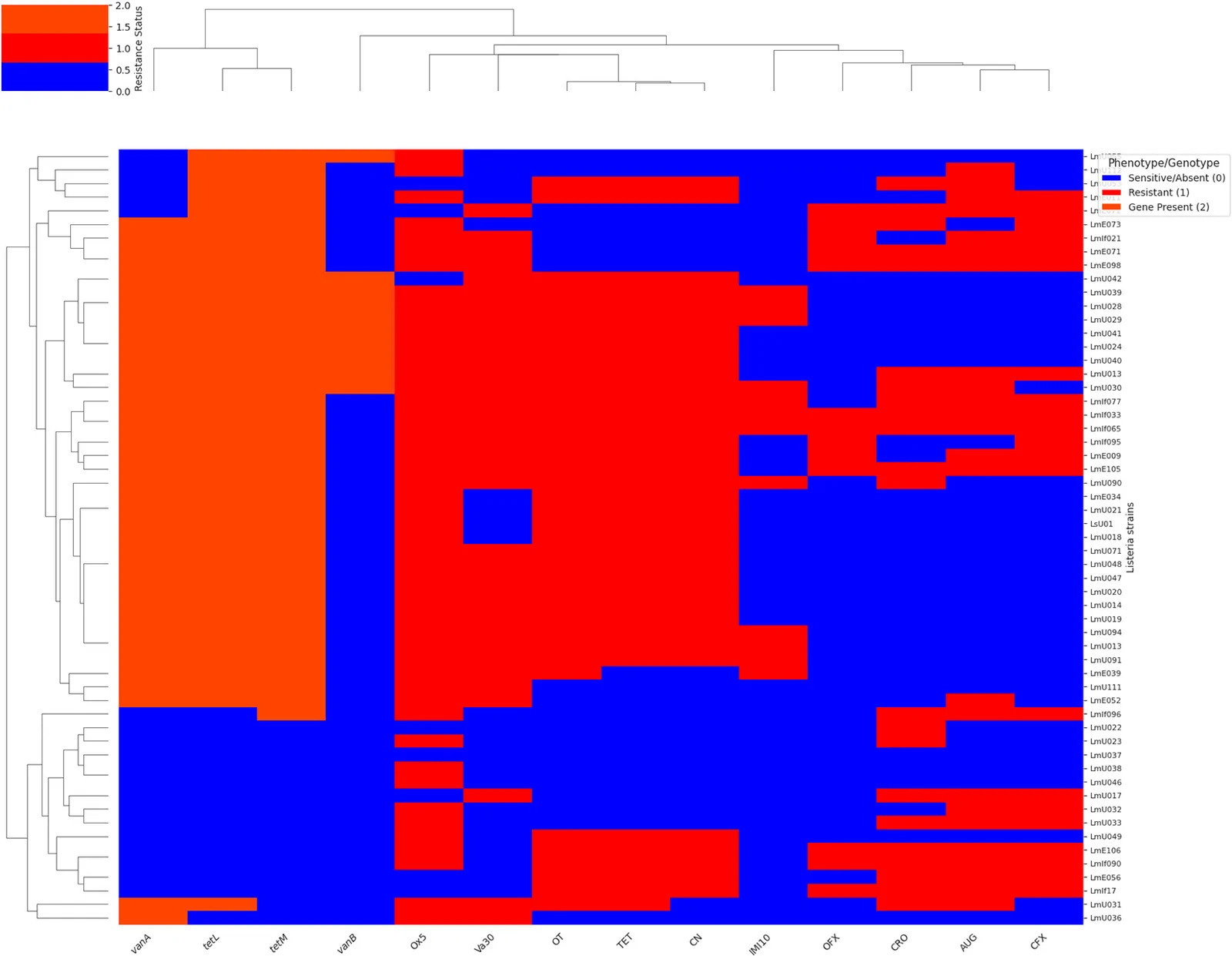
Hierarchical clustering heatmap of antibiotic resistance vs selected antibiotic resistance genes among selected strains.

Figure 4 presents a hierarchical clustering heatmap illustrating the antibiotic resistance profiles and the presence of specific antibiotic resistance genes in a collection of *Listeria* strains. The heatmap is coloured to indicate sensitive/absent (blue, 0), resistant (red, 1), and gene presence (reddish-orange, 2) for each strain and antibiotic/gene. Hierarchical clustering was applied to both the *Listeria* strains (rows) and the antibiotic/gene markers (columns) to group those with similar patterns of resistance and gene presence, as depicted by the dendrograms on the left and top of the heatmap, respectively. The x-axis labels include the names of antibiotics and italicised names of resistance genes, while the y-axis labels represent the individual *Listeria* strains. This visualisation allows for the identification of clusters of strains with similar resistance phenotypes and genotypes, providing insights into the relationships between antibiotic resistance and the presence of these specific genes within the *Listeria* collection.

### 3.5 Correlation assessment of the antibiotic resistance with antibiotic resistance genes

**Figure 5.**
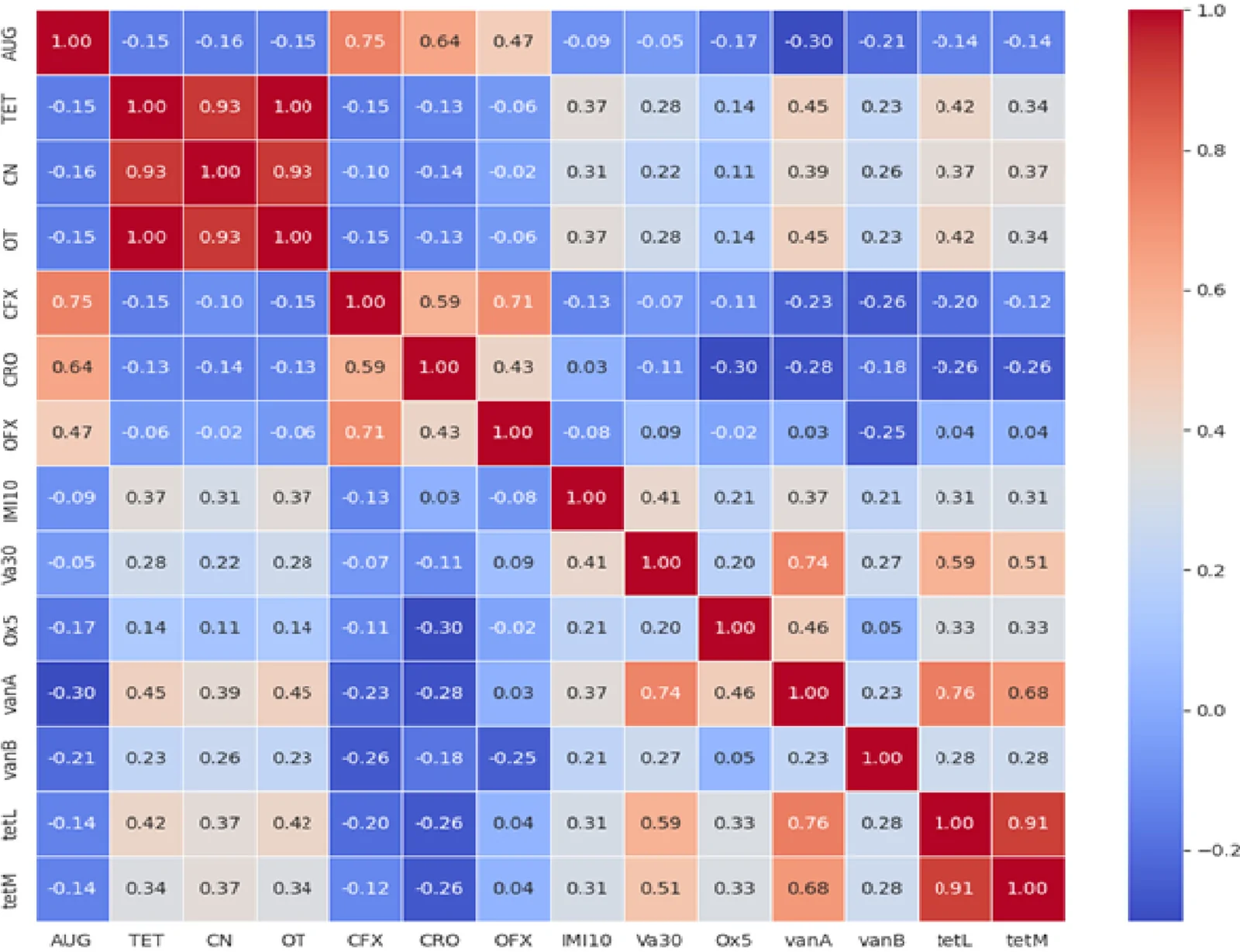
Correlation Matrix of the Antibiotic Resistance with Antibiotic Resistance Genes.

Figure 5 presents a heatmap visualising the pairwise correlations between the antibiotic resistance profiles and the presence of specific resistance genes. The heatmap utilises a colour scale to represent the strength and direction of the correlation coefficients, ranging from strong negative correlations (cool colours) to strong positive correlations (warm colours). Each cell in the heatmap displays the calculated Pearson correlation coefficient between the corresponding antibiotic/gene pairs. For example, AUG (augmentin) shows a positive correlation with CRO (ceftriaxone) with a coefficient of 0.64, suggesting that resistance to AUG is often associated with resistance to CRO in this dataset. Conversely, AUG exhibits negative correlations with the antibiotic resistance genes *vanA* (-0.30), *vanB* (-0.21), *tetL* (-0.14), and *tetM* (-0.14), indicating that resistance to AUG is generally less likely when these specific genes are present. This visualisation provides a clear overview of the interdependencies among the measured resistance phenotypes and genotypes, enabling the identification of highly correlated variables that may suggest potential associations between specific resistance mechanisms and observed resistance patterns.

### 3.6 Quantitative microbial risk assessment

The results of this QMRA are presented in Table 4. The table provides a comprehensive overview, detailing the produce type, region, sample size, original enumeration data from both culture media, the calculated mean count, and the resulting risk estimates. The data reveal significant variations in the estimated risk, differing by over two orders of magnitude between the highest and lowest risk-producing items. This stratification of risk directly correlates with the microbial load data, effectively identifying high-risk produce-region combinations that warrant targeted intervention strategies.

**Table 4.** Quantitative microbial risk assessment results.

Produce type	Region	No of samples	Oxford_CFU_25g	Microinstant_CFU_25g	Mean_CFU_25g	Dose_per_serving_CFU	P_ill_per_serving	P_ill_annual
Water Leaf	South South (Akwa-Ibom)	21	3.25e+02	2.60e+02	2.92e+02	1.17e+03	2.77e-11	1.44e-09
Water Leaf	South West (Ile-Ife)	18	2.50e+01	5.00e+01	3.75e+01	1.50e+02	3.55e-12	1.85e-10
Cabbage (kabeji)	North (Kano)	9	6.75e+02	5.40e+02	6.08e+02	2.43e+03	5.76e-11	2.99e-09
Carrot	North (Bauchi)	30	5.70e+02	4.50e+02	5.10e+02	2.04e+03	4.83e-11	2.51e-09
Pumpkin	North (Kano/Bauchi)	11	2.50e+01	2.00e+01	2.25e+01	9.00e+01	2.13e-12	1.11e-10
Lady finger (Mata yatsa)	North (Kano/Bauchi)	7	9.25e+02	7.40e+02	8.32e+02	3.33e+03	7.89e-11	4.10e-09
Lettuce (lecttuse)	North (Kano/Bauchi)	11	5.50e+02	5.50e+02	5.50e+02	2.20e+03	5.21e-11	2.71e-09
Amaranth	North (Kano)	9	8.25e+02	6.60e+02	7.42e+02	2.97e+03	7.04e-11	3.66e-09
Ugwu	South East (Enugu)	11	2.42e+02	1.94e+02	2.18e+02	8.72e+02	2.07e-11	1.07e-09
Ora	South East (Enugu)	9	2.50e+01	2.00e+01	2.25e+01	9.00e+01	2.13e-12	1.11e-10
Onugbu	South East (Enugu)	10	2.25e+02	1.80e+02	2.02e+02	8.10e+02	1.92e-11	9.98e-10
Nchuanwu	South East (Enugu)	9	1.75e+02	1.40e+02	1.58e+02	6.30e+02	1.49e-11	7.76e-10
Garden egg	South West (Ile-Ife)	30	2.10e+02	1.75e+02	1.92e+02	7.70e+02	1.82e-11	9.49e-10
Water Melon	South South (Akwa-Ibom)	15	2.50e+01	3.00e+02	1.62e+02	6.50e+02	1.54e-11	8.01e-10
Water Melon	South West (Ile-Ife)	8	1.25e+02	1.00e+02	1.12e+02	4.50e+02	1.07e-11	5.55e-10

## 4. Discussion

This study provides an assessment of the prevalence, load, genetic diversity, antimicrobial resistance (AMR), and associated health risks of *Listeria monocytogenes* in fresh fruits and vegetables across Nigeria’s major geopolitical regions. The findings paint a concerning picture of widespread contamination with potentially pathogenic and resistant strains, necessitating urgent and targeted interventions within the national food safety system.

The overall *Listeria* species prevalence of 38.5% observed in this study is substantially higher than rates reported in similar surveys from many other regions. For instance, studies in South Africa ^29^ and Morocco ^30^ reported prevalence rates of 15% and 12.5% on fresh produce, respectively. This disparity likely reflects critical differences in agricultural and post-harvest practices. The elevated contamination levels, particularly in Northern regions (Kano and Bauchi), strongly suggest a linkage to the widespread use of untreated wastewater or polluted surface water for irrigation, a common practice in water-scarce regions of Nigeria and many other LMICs. ^7^ The high bacterial loads found on vegetables like Lady’s finger and Amaranth (exceeding 8.0 × 10 ^2^ CFU/25g) are particularly alarming, as these are often consumed with minimal processing, thereby bypassing potential microbial reduction steps.

The molecular characterisation confirmed the predominance of *L. monocytogenes* among the isolates. The phylogenetic analysis revealed significant genetic diversity, with strains clustering into distinct clades. The deposition of numerous *de novo* sequences (e.g., PX282429.1 - PX282453.1) indicates the presence of novel, locally circulating strains, while other isolates showed high identity (up to 99.15%) to global reference strains. This genetic heterogeneity suggests multiple and possibly ongoing sources of contamination, including environmental reservoirs and cross-contamination points along the supply chain, from farm to market. ^31^ The presence of both unique and globally disseminated clones underscores the dual challenge of managing local food safety hazards while remaining vigilant against the introduction of internationally prevalent pathogens.

A central and critically important finding of this work is the high prevalence of antimicrobial resistance among the *Listeria* isolates. The alarming resistance rates to vancomycin (83.1%) and oxacillin (88.1%)—both critically important antibiotics for human medicine—pose a severe threat to public health. While intrinsic resistance to some antibiotics is known in *Listeria*, the high frequency of resistance to these specific agents is deeply concerning. ^32^ Vancomycin resistance may also be a testament to induced resistance resulting from the presence of antibiotics and other emerging contaminants in the organic fertiliser or wastewater. ^32– 35^ These findings are consistent with a growing body of evidence from LMICs indicating the emergence of multi-drug resistant (MDR) *Listeria* in the food chain, likely driven by the selective pressure from the misuse of antibiotics in agriculture and human medicine. ^36^ The hierarchical clustering heatmaps effectively identified clusters of MDR strains, revealing co-resistance patterns that could complicate treatment options. The detection of resistance genes ( *vanA, vanB, tetL, tetM*) in some isolates, though not always perfectly correlated with the phenotypic resistance, confirms the genetic potential for resistance dissemination. The correlation matrix further elucidated complex relationships, such as the positive correlation between AUG and CRO resistance, hinting at possible co-selection mechanisms. The presence of the *hly* virulence gene in pathogenic strains confirms their potential to cause disease, making the concomitant AMR profile a dangerous combination.

The Quantitative Microbial Risk Assessment (QMRA) model translated microbial contamination data into tangible public health risk estimates. Although the calculated annual probability of illness per individual appears low (on the order of 10 ^−9^ to 10 ^−10^), this risk must be interpreted at the population level. Given Nigeria’s large population and the high frequency of consumption of these commodities, even a low per-serving risk can translate into a significant number of annual illnesses. Furthermore, the model was built on conservative assumptions (e.g., 100% prevalence of contamination, no reduction factor), representing a worst-case scenario that is crucial for informing protective food safety policies. The risk stratification successfully identified high-risk produce-region combinations (e.g., Lady’s finger and Amaranth from the North), providing a clear evidence-based roadmap for prioritising intervention efforts.

The findings of this study carry profound and immediate implications for food safety governance in Nigeria and offer a cautionary model for other regions with similar agricultural practices. The high prevalence and load of *Listeria monocytogenes*, coupled with its concerning antibiotic resistance profile and proven pathogenicity, necessitate a fundamental shift from reactive to proactive, evidence-based food safety plans. First and foremost, the data provides an incontrovertible case for the urgent prioritisation of agricultural water safety. The significantly higher contamination levels in produce from regions like Kano and Bauchi strongly implicate the use of contaminated irrigation water as a critical control point. National policy must, therefore, move towards the formal adoption and enforcement of guidelines for the safe use of wastewater in agriculture, as outlined by the World Health Organization. ^37^ This requires a multi-sectoral effort involving the Ministry of Water Resources and the Ministry of Agriculture to promote and subsidize low-cost, on-farm water treatment solutions, such as ponding or filtration systems, and to map and monitor water sources used for irrigation near urban centers.

Furthermore, the alarming prevalence of antimicrobial resistance (AMR), particularly to critically important antibiotics like vancomycin, demands the immediate integration of foodborne pathogen surveillance into the national AMR action plan. The discovery of multi-drug resistant (MDR) *L. monocytogenes* strains in the food chain represents a direct threat to public health, potentially rendering life-saving treatments ineffective in cases of severe listeriosis. Food safety policies must now explicitly link agricultural practices with the AMR crisis. This involves strengthening regulations on the use of medically important antibiotics in livestock and crop production to reduce selective pressure. Moreover, the national food safety authority, in collaboration with public health institutes, must establish a structured program for the routine monitoring and genomic surveillance of AMR in key foodborne pathogens isolated from food, animals, and the environment, creating an integrated “One Health” surveillance database. ^38^

Finally, the Quantitative Microbial Risk Assessment (QMRA) model employed in this study offers a powerful template for evidence-based policy and targeted intervention. The risk characterisation, which clearly identifies high-risk produce (e.g., Lady’s finger, Amaranth) from high-risk regions, allows for the efficient allocation of limited resources. Food safety plans can now be tailored specifically to these commodities, focusing on their supply chains. This could include mandatory certification of irrigation water quality for farms growing these high-risk crops, enhanced Good Agricultural Practices (GAP) training for these farmers, and targeted point-of-sale consumer advisories. For the wider market, the policy should mandate and incentivise the adoption of improved post-harvest handling, storage, and transportation infrastructures to maintain cold chains and prevent cross-contamination. Ultimately, this study provides the scientific foundation to transition from generic food safety guidelines to a targeted, risk-based regulatory framework that protects consumers, supports responsible farmers, and safeguards the efficacy of antimicrobials for future generations.

## 5. Conclusion

This comprehensive study provides a critical, evidence-based assessment of the contamination of fresh fruits and vegetables with *Listeria monocytogenes* in Nigeria, revealing significant public health risks that demand immediate and coordinated action. The high prevalence (38.5%) and substantial microbial loads of *Listeria* species, particularly in produce from the Northern regions, highlight a widespread issue of contamination, most likely originating from the use of untreated animal manure and polluted water for irrigation. The molecular characterisation confirmed the presence of diverse strains, including novel local variants and others closely related to global clones, underscoring a complex ecology of this pathogen within the Nigerian agricultural landscape.

The finding of a high prevalence of antimicrobial resistance among the isolates, especially to critically important antibiotics such as vancomycin, is probably a result of plasmid-based van genes transferred from Enterococci. The presence of resistance genes and the identification of multidrug-resistant clusters signal a troubling convergence of virulence and resistance, which could severely compromise treatment options for listeriosis. The quantitative microbial risk assessment successfully translated these microbiological findings into a public health context, identifying high-risk produce and regions and providing a scientific basis for prioritising interventions.

Finally, the results of this study serve as a stark warning and a clear call to action. Fresh produce, a cornerstone of a healthy diet, is acting as a vehicle for pathogenic and resistant *L. monocytogenes* strains in Nigeria. Addressing this threat necessitates a paradigm shift towards a proactive, evidence-based, and multi-sectoral “One Health” approach. This includes implementing strict agricultural water safety policies, integrating foodborne pathogen surveillance into national antimicrobial resistance action plans, and launching targeted education programs for producers and consumers. The findings presented herein are not merely academic; they provide the essential scientific foundation for policymakers, regulatory bodies, and public health officials to develop robust and targeted strategies to safeguard food security and public health in Nigeria and other regions facing similar challenges.

## Ethical considerations

On this study on *Listeria* in Nigerian produce, it is confirmed that all ethical protocols were strictly followed. Vendor and farm anonymity were maintained, and significant findings on antibiotic resistance were communicated to public health authorities to guide food safety policy without causing undue public alarm. All laboratory analyses adhered to rigorous biosafety standards throughout the research.

## Data Availability

The nucleotide sequence data generated in this study have been deposited in the National Center for Biotechnology Information (NCBI) GenBank database. The sequences are publicly accessible under the accession numbers PX282429.1 through to PX282453.1. This repository ensures the permanent availability and integrity of the data for future research.
