# Supplementary material for: Antibiogram, antibiotic resistance genes and health risks of Listeria species from fruits and vegetables in selected locations in Nigeria
Source: F1000Res. 2026 Jun 23;14:1421. Originally published 2025 Dec 22. [Version 2] doi: 10.12688/f1000research.171697.2 (PMC13555043; doi:10.12688/f1000research.171697.2)
Supplement: Peer review history

# Reviewer report: Reviewer response for version 2

**DOI:** 10.5256/f1000research.198761.r496977

**Published:** 2026-07-24

**Authors:** Joanna G Rothwell Rothwell

**Affiliations:** 1 University of California, Davis, USA

**Recommendation: Major Revision**

 This article is an important area of research, and I strongly agree with the authors in that more research is needed in Nigeria in terms of foodborne disease pathogen transmission dynamics.

**General: **

- Make sure *Listeria* is in italics throughout the manuscript.
- More information should be in the table and figure legends so that the data can be interpreted without reading the whole text.
- In general, the article is long and would benefit from more concise writing in the methods and results.
- The limit of detection is critical and should be listed in the table 2 and methods.
- The results sections need more emphasis on what were the results and less explanation on what the tables or figure are. Report the data that is most important in the text.
- The authors must explicitly state the exact number and percentage of samples (and total isolates) that were definitively identified as *L. monocytogenes. *The current manuscript conflates genus-level Listeria spp. findings with the pathogenic species Listeria monocytogenes.
- Amend over-interpretation of 16S rRNA sequencing data
- Report intermediate values as well as resistant values.
- There is no table 3B
- More reference to other studies in the discussion re virulence and AMR genes would be beneficial.

**Abstract:**

- In methods, list what the ‘conventional microbiological techniques and molecular methods’ are.
- List the number of regions and total farms included in the study
- Include the risk assessment in the methods

**Results: **

- What do the authors mean by: ‘others closely related to global clones’?  16S rRNA sequencing lacks the resolution required for clonal identification, strain typing, or inferring environmental resilience in *L. monocytogenes.*
-  If space allows, report the prevalence of detection of resistance genes and virulence genes.

**Introduction:**

 Introduction is well written. In the AMR paragraph, please explain what drugs are used in treatment of listeriosis, cite other studies where your selected resistance genes in Listeria have been identified previously as concerning and highlight why they are important to assess in this study ( *vanA, vanB, tetL, tetM*).

**Typo:**

 We quantify —> The study aims are to quantify..

 molecularly characterize —> reword

**Materials and methods:**

- In general, the methods section needs to be more concise as sections are over explained.
- ‘Within each region, sampling points were strategically selected to encompass major farms known to utilise animal waste manure for fertilisation and wastewater or polluted water sources for irrigation purposes.’ Does this bias the dataset towards worse outcomes? I suggest adding this detail into the abstract and discussion as it changes the direction of the study.
- ‘About 30 samples were collected from farms not using such manure, because there were cow droppings within and around the farmland (inadvertent manuring), which obviously were expected to have impacts on the crops.’
- This is not clear to the reader what was done.
- ‘“No Positive” refers to the number of composite samples that yielded *Listeria* growth’. Suggest to relabel #positive or n=positive or % positive or just say number positive.

**Needs rewording:  **

- For Southwest analysis, pre-enriched incubation in serially diluted Half-Fraser Broth and Full-Fraser Broth (OXOID, CM0895 ISO, United Kingdom) at 37 oC for 24-48 h. 
- Section 2.3 could be just added to the previous section 2.2 Specify what plates the purification was done on and how were the isolates stored, in glycerols in -80C?
- 2.6.1: remove “a facultative anaerobic, Gram-positive bacterium known to cause the severe foodborne illness listeriosis.”
- 2.7 Typo: turbidity equivalent to 0. 0.5 McFarland standard,
- I disagree with reporting intermediate isolates as resistant. For the risk assessment maybe but for reporting the resistance rates you should include the number of intermediate isolates. You can include this in a squashtogram for easy reporting.
- Listeria not in italics
- Shorten this or remove it: “The overall resistance prevalence was depicted using a simplified stacked bar chart, which clearly illustrated the percentage of isolates classified as resistant or sensitive for each antibiotic, providing an immediate macroscopic view of resistance trends.”
- This also needs to be shortened and this information should be in the figure legends.
- “To explore the patterns of multidrug resistance and relationships between the isolates, a hierarchical clustering heatmap was constructed. This analysis employed the Jaccard distance metric and average linkage method to cluster both the bacterial isolates based on their antibiograms and the antibiotics based on their co-resistance patterns. This dual dendrogram heatmap effectively identified clusters of multidrug-resistant strains and groups of antibiotics to which resistance was commonly linked, offering a sophisticated visual representation of the complex susceptibility profile data.”
- 2.8  Remove: which is a well-characterised and specific protocol for identifying this critical virulence factor in *Listeria monocytogenes.*
- Shorten the PCR methods section.
- Table 2, remove the scientific notation, the numbers are so low it is not particularly helpful. Include the min and maximum values not just 3 averages.

**Results:**

- Typo in 3.1 Quantification and isolation, occurrence and frequencies of occurrence of *Listeria* species
- Remove this: The table presents the average bacterial counts, expressed in colony-forming units per 25 grams (CFU/25 g), obtained from two selective culture media: Oxford Listeria (Oxoid) and Microinstant® *Listeria* (Scharlab). The final composite average count for each produce-type and region combination was calculated from these values.
- Table 3. (a) and (b): Bacterial identities, sources and accession number. There is only 1 table, the source (location) and food type is not listed. Include this information along with the year isolated or date.
- Figure 1: remove the 16S ribosomal RNA gene partial sequence from the figure
- Figure 2 Is very distorted and I would suggest presenting this data in a table not a bar chart or use a squashtogram.
- 3.4 Remove this: This figure offers a clear visual summary of the resistance patterns observed in the tested *Listeria* isolates. While Figure presents the antibiotic profile, other subsequent figures present the pattern and relationships with other factors.
- Figure 3: legend is blocking the listeria names. Figure is unclear.
- This is not a result, it is explaining the figure, which should be in the figure legend not in the main text:
- “ Figure 3 displays a hierarchical clustering heatmap that visualises the antibiotic resistance profiles and the presence of specific resistance genes across the *Listeria* strains analysed. The heatmap employs a colour gradient where blue represents sensitive or gene absence, red indicates resistance, and reddish-orange signifies the presence of a resistance gene. The dendrograms flanking the heatmap illustrate the hierarchical clustering of both the *Listeria* strains (rows) and the antibiotic/gene markers (columns), highlighting groups with similar patterns. The x-axis is labelled with the antibiotic names and italicised resistance gene names, while the y-axis shows the individual *Listeria* strains. This figure facilitates the identification of correlations between observed antibiotic resistance phenotypes and the presence of the investigated resistance genes, revealing potential relationships and clustering patterns within the *Listeria* isolates.”
- Same applies for Fig. 4 and Fig 5.
- Section 3.7 needs more reporting of the results not explaining what is in the table. It is not clear what the findings were.

**Discussion:**

- "The findings paint a concerning picture of widespread contamination with potentially pathogenic and resistant strains, necessitating urgent and targeted interventions within the national food safety system." this sentence needs to also list that the farms were using the untreated animal manure and untreated irrigation water- findings cannot be generalised to all farms.
- Authors have over-interpretated the capacity of 16S rRNA resolution. Please revise and correct this discussion paragraph. The claims would only be supported with WGS/MLST.
- Listeria monocytogenes has intrinsic/natural low susceptibility to oxacillin. Penicillin, ampicillin, and amoxicillin are frequently used for treating Listeria infections, these would be more valuable to discuss here.

**Conclusions:**

 Remove assertions regarding diverse strains, and global clones. Standard 16S rRNA gene sequencing cannot resolve strain-level variation or clonal lineages in *Listeria*.

 Report the total number of L. mono here not just total listeria isolates as not all listeria are pathogenic.

Is the work clearly and accurately presented and does it cite the current literature?

Partly

If applicable, is the statistical analysis and its interpretation appropriate?

Partly

Are all the source data underlying the results available to ensure full reproducibility?

Yes

Is the study design appropriate and is the work technically sound?

Yes

Are the conclusions drawn adequately supported by the results?

Partly

Are sufficient details of methods and analysis provided to allow replication by others?

Yes

Reviewer Expertise:

NA

I confirm that I have read this submission and believe that I have an appropriate level of expertise to confirm that it is of an acceptable scientific standard, however I have significant reservations, as outlined above.

# Reviewer report: Reviewer response for version 2

**DOI:** 10.5256/f1000research.198761.r496981

**Published:** 2026-07-23

**Authors:** Hugo Guedes, Gonçalo Almeida

**Affiliations:** 1 Nacional Institute for Agrarian and Veterinary Research, Instituto Nacional de Investigação Agrária e Veterinária, I.P., Rua dos Lagidos – Lugar da Madalena, Vairão, Portugal; 2 Food Microbiology, Nacional Institute for Agrarian and Veterinary Research, Instituto Nacional de Investigação Agrária e Veterinária, I.P., Vairão, VCD, Portugal

**Comments**

 Nomenclature should be revised. Listeria should be written the first time it appears. After that please use L. monocytogenes an always in italic.

 Abstract - Results section

 The statement that the isolates were "genetically distinct" and "closely related to global clones" is stronger than the data support. Only partial 16S rRNA sequences were analysed, which do not provide sufficient discriminatory power for this level of inference.

**1. Introduction**

 Authors wrote “A critical characteristic of L. monocytogenes is its psychrotrophic nature, enabling it to survive and multiply at refrigeration temperatures, which challenges conventional food preservation strategies and extends its shelf-life within the food chain” – I think it should be re-write. What extends shelf-life?

**2. Materials and Methods**

**Section 2.1 - Study areas and sample collection**

*Paragraph 2*

 The poolings are of the same matrix or different matrices? If you have a positive result how can you detect which farm or vendor is responsible for the contamination? The pooling of samples inhibits the rastreability to the product of farmer.

**Section 2.2 – Listerial quantification and isolation procedure**

 Quantification of Listeria spp. was performed using a standard plate count technique following a ten-fold serial dilution. Briefly, 25 g of each composite sample was homogenised in 225 mL of Buffered Peptone Water (BPW)” – that means you performed 1:10 dilution so the results obtained are /g not /25g. That means that you should review you results and Quantitative Microbial Risk Assessment.

*Paragraph 2*

 The description of the Southwest protocol is unclear. The manuscript mentions pre-enrichment in Half-Fraser and Full-Fraser broth, whereas direct plating is described for the remaining samples. Please clarify whether the reported CFU/25 g values were obtained before or after enrichment, as enrichment cannot be used for quantitative enumeration. Have you used an ISO standard? Is this method validated? Which is the reference for the medium?

*Paragraph 3*

 The authors refer “The mean value for enumeration was obtained using the result of the 2 media”. Did you used the data obtained from 2 complete different medium? The 2 media has the same productivity?

 For correct enumeration the mean has always to be obtained from parallel enumerations of the same media.

**Section 2.3 - Purification and subculture of isolates for characterisation**

 The agar plates  for subculturing where on selective media, nutrient media, blood agar media?

**Section 2.4 Phenotypic identification of Listeria spp.**

 Nowadays some *L. mono* present α-haemolysis. You had ß- haemolysis in all isolates? Is there any possibility of false negative because of the haemolysis results? No table was presented. Please clarify.

**Section 2.5 – Molecular identification**

*Paragraphs 2.5.2–2.5.6*

 Species identification relies exclusively on partial 16S rRNA sequencing. This marker has limited discriminatory power for Listeria monocytogenes and should not be used to infer strain diversity or epidemiological relationships.

 Are the primers used  from literarure or designed by the laboratory? Please clarify.

**Section 2.7 – Antibiotic susceptibility profiling**

 Please clarify the antimicrobial susceptibility results. Seven antibiotics are described in the Methods, whereas Figure 2 includes ten, and some percentages reported in the text do not match those shown in the figure. The authors should review these results and provide the underlying inhibition-zone and MIC data.

 Is the Python program deposited in any repository like Github? Is yes it must be referred. The primer concentration must be referred on a table for future readers has the opportunity to use this method.

**Section 2.8 - Detection of antibiotic and virulence genes**

 The protocol used was adapted, it was made the proper validation? Annealing temperature adjustment, agreement of the results between the 2 methods?

**3. Results**

**Section 3.1 - Quantification and isolation, occurrence and frequencies of occurrence of Listeria species**

 The number of samples is not the same per zone, so the incidence of positives could be different. If in one place you collect 21 samples of Water Leaf from one place and you collect 23 samples of water melon but from 2 different regions it's difficult to relate the results only by the number of samples. First you are comparing 2 different matrices with 2 different productions methods. We are relating 3 different places where the contamination of soils, water or workers behaviour can influence results. I suggest a different approach on data treatment.

**Section 3.2 - Isolated Listeria identity**

 For this part of the study you should refer the software’s used and versions.

**Section 3.3 - Phylogenetic tree**

 The use of a phylogenetic tree from a partial 16S rRNA gene sequence is not appropriate for comparing Listeria monocytogenes isolates, as this marker lacks sufficient sequence variability to resolve intraspecific relationships. Due to the highly conserved nature of the 16S rRNA gene, this approach is generally suitable for genus/species identification but is not appropriate for assessing phylogenetic relationships among L. monocytogenes isolates or for inferring epidemiological relatedness. The authors should either justify the use of this marker and acknowledge its limitations or consider employing higher-resolution genomic approaches (e.g., MLST, cgMLST or whole-genome SNP analysis).

**Table 2  - Enumeration of Listeria species Fruit and Vegetable Samples**

 The numerical data in Table 2 requires verification against the raw dataset. The reported numbers of positive samples sum to 79, not 80, corresponding to an overall occurrence of approximately 38.0% rather than 38.5%. Several row percentages and mean counts are also arithmetically inconsistent. These errors affect the Results, Discussion and Conclusion.

 Please present the results with only one decimal.

**Table 3 - (a)and(b): Bacterial identities, sources and accession number**

 The species assignments in Table 3(b) require verification. Some isolates are reported as L. monocytogenes despite only 88.89–96.20% 16S rRNA sequence identity to the listed references. Such values do not support confident species-level identification and may indicate poor sequence quality, incorrect alignment, contamination or misinterpretation of BLAST results. Sequence length, query coverage, number of ambiguous bases and identity to validated type/reference strains should be reported.

**Figure 1- Phylogenetic relationships of the Listeria species from selected Nigerian locations**

 The phylogenetic tree is difficult to interpret due to its presentation. The figure does not clearly convey the genetic differences among the isolates, making it difficult for the reader to assess the biological significance of the observed clustering. A clearer and more informative representation is recommended.

**Figure 3 – Hierarchical clustering heat map of scaled antibiotic resistance profiles and *hly* genes across selected *Listeria* strains.**

 Figure 3 is difficult to interpret due to its presentation. Although a hierarchical clustering tree is included, the relationship between the dendrogram branches and the corresponding isolates is not readily apparent. Consequently, it is difficult for the reader to identify which isolates belong to each cluster and to relate the clustering to the antibiotic resistance and gene profiles. The figure should be reformatted to improve readability and facilitate the interpretation of the clustering results.

**Section 3.4 – Antibiogram of the Listeria species**

 The antimicrobial susceptibility results requires revision. The Methods describe seven antibiotics whereas the figure contains ten variables. The percentages reported in the text should also be checked for consistency with the figure.

 It is described an intrinsic resistance of L. monocytogenes to cephalosporines. The evaluation of resistance should be performed carefully.

 Section 3.5 - Hierarchical clustering heatmap of scaled antibiotic resistance and virulence profiles across the Listeria strains

 The heatmaps are based on 7 most expected antibiotics and confirmation using phenotypique analyses. Some other genes can be present but without phenotypique expression and others can be expressed but not found. A WGS approach is suggested to support this subject.

**Figure 4**

 The same limitation applies to Figure 4. The dendrogram does not allow the reader to identify which isolates belong to each cluster, making biological interpretation difficult. Differences are not visually detected.

**4. Discussion**

 “Meanwhile, we acknowledge that composite sampling provides robust average contamination estimates but limits inference about true individual-sample prevalence

 and variability. These values are still of overall epidemiological importance. Paragraph discussing molecular diversity” – What if one sub-sample was highly contaminated?

 Please include a definition of multi drug resistance.

 “The presence of the hly virulence gene in pathogenic strains confirms their potential to cause disease, making the concomitant AMR profile a dangerous combination” – hly is part of the LIPI-1 genes and is normally present in L. monocytogenes. Please change the sentence.

 The manuscript attributes "significant genetic diversity" and "locally circulating strains" to the phylogenetic analysis. These conclusions are not supported by partial 16S rRNA sequencing and should be substantially moderated.

*Paragraph 2*

 Reference 35 (Marocco study) - This study is not on fresh produce but on beef and chicken carcasses and the results can not be related.

 On the other hand, The bibliographic information provided in references 34 and 35 does not correspond to the cited studies. Specifically, PLOS ONE article e0251757 is a correction related to a palliative care study, while Journal of Food Safety article e12605 concerns ESBL-producing Escherichia coli in Turkey. Therefore, these references do not support the statement regarding the prevalence of Listeria monocytogenes in fresh produce from South Africa and Morocco and should be carefully verified.

 The conclusions drawn from the 16S rRNA phylogenetic analysis appear to be overstated. A partial 16S rRNA sequence does not provide sufficient discriminatory power to infer significant genetic diversity, define distinct phylogenetic clades, or conclude the presence of genetically distinct locally circulating strains of L. monocytogenes. The discussion should be revised to better reflect the limitations of the marker and avoid overinterpretation of the phylogenetic results.

*Paragraph 3*

 Reference 37 - The cited reference does not support the conclusion that the observed 16S rRNA diversity indicates multiple contamination sources. This statement appears to overinterpret the results and should be revised.

**5. Conclusion**

*“The molecular characterisation confirmed the presence of diverse strains, including novel local variants and others closely related to global clones, underscoring a complex ecology of this pathogen within the Nigerian agricultural landscape.” *The authors cannot conclude this only using parcial 16s rRNA gene.

**References**

 Several references should be checked and correct

 Statements regarding genetic diversity and strain circulation should rely on studies using high-resolution typing methods (MLST, cgMLST or WGS), rather than partial 16S rRNA sequencing.

Is the work clearly and accurately presented and does it cite the current literature?

Partly

If applicable, is the statistical analysis and its interpretation appropriate?

Partly

Are all the source data underlying the results available to ensure full reproducibility?

No

Is the study design appropriate and is the work technically sound?

No

Are the conclusions drawn adequately supported by the results?

No

Are sufficient details of methods and analysis provided to allow replication by others?

No

Reviewer Expertise:

Food Microbiology and WGS

We confirm that we have read this submission and believe that we have an appropriate level of expertise to state that we do not consider it to be of an acceptable scientific standard, for reasons outlined above.

# Reviewer report: Reviewer response for version 1

**DOI:** 10.5256/f1000research.189332.r460403

**Published:** 2026-03-05

**Authors:** Bahar Onaran Acar

**Affiliations:** 1 Ankara University, Ankara, Turkey

This manuscript reports a multi-region survey of *Listeria* spp. contamination in fresh fruits and vegetables collected across major geopolitical regions of Nigeria. The authors enumerate *Listeria* loads (CFU/25 g) using selective media, identify isolates using 16S rRNA sequencing with phylogenetic analysis, characterize antimicrobial susceptibility by disk diffusion, detect selected resistance and virulence genes, and integrate these data into a Quantitative Microbial Risk Assessment (QMRA) to estimate annual probability of illness. The study reports an overall occurrence rate of 38.5% and highlights high contamination in some produce-region combinations, with the manuscript emphasizing high reported resistance to vancomycin and oxacillin, detection of resistance genes and *hly*, and a QMRA framework based on conservative assumptions. 

**1) Is the work clearly and accurately presented and does it cite the current literature?**

**Answer: Partly**

**Strengths**

- The manuscript is generally well organized (Introduction → Methods → Results → Discussion → Conclusion) and explains the intention to integrate microbiological data with QMRA and AMR characterization.
- Key methodological steps (sampling, enumeration, disk diffusion workflow, QMRA equations) are described in a way that is broadly understandable.

**Concerns and constructive recommendations**

- **Recency and balance of citations**: Some core background citations are older (e.g., general *Listeria* reviews). This is not necessarily incorrect, but AMR and produce-chain risk literature evolves quickly. The authors should add several recent references (last ~5–7 years) on:
  - *Listeria* AMR surveillance in produce and LMIC contexts,
  - validity/limitations of disk diffusion and breakpoint interpretation for *Listeria*,
  - contemporary QMRA best practices (uncertainty/sensitivity analysis; parameter justification for prevalence, reduction factors, and susceptible subpopulation fractions).
- **Overstatement of “novel strains”**: The manuscript links GenBank deposits and “de novo” sequences to novelty (e.g., “indicates the presence of novel, locally circulating strains”).

With 16S rRNA sequencing alone, “novel strain” claims should be toned down unless supported by WGS-based typing (cgMLST/SNP) and clear novelty criteria.

- **Language alignment with risk metrics**: The paper frames a “serious public health threat” while also acknowledging very low annual illness probabilities.

This can be reconciled, but the discussion must carefully explain “individual risk vs population burden” and avoid escalation beyond what the model supports.

**2) Is the study design appropriate and is the work technically sound?**

**Answer: Partly**

**Strengths**

- Multi-regional sampling strategy provides useful geographic coverage.
- Combining enumeration + molecular ID + AMR phenotyping/genotyping + QMRA is a meaningful “farm-to-fork-to-risk” structure.

**Major concerns and constructive recommendations**

1. **Composite sampling design limits prevalence inference**

- The study creates composite samples by pooling sub-samples from ≥5 points per produce type per location.

This approach can estimate an “average contamination level,” but it **reduces the ability to estimate true prevalence**, variability, and uncertainty at the unit level (farm/vendor/sample).

**How to address**: Explicitly state this limitation and avoid interpreting “% positive” as a typical individual-sample prevalence unless the unit of analysis is clearly the composite. If possible, provide the number of composites and clarify how “No Positive” was computed for pooled samples.

1. **Antibiotic susceptibility methodology—breakpoint validity**

- The authors state they applied cefoxitin and oxacillin breakpoints from *Staphylococcus* spp. because *Listeria*-specific criteria are unavailable for all antibiotics.

This may lead to **misclassification of resistance** and can inflate headline resistance rates.

**How to address**:

1.
  - Provide a table listing each antibiotic, the guideline/source, and the exact breakpoints used.
  - Where no validated *Listeria* breakpoints exist, consider removing those antibiotics from “clinical” resistance claims or clearly labeling them as “non-standard interpretive criteria used for exploratory comparison.”
  - Prefer MIC testing (broth microdilution or E-test) for critical drugs where resistance is unexpected/high (see next point).
2. **Extraordinary vancomycin resistance claim needs confirmatory testing**

- The manuscript highlights very high vancomycin resistance (83.1%) and oxacillin resistance (88.1%).

Such unusually high vancomycin non-susceptibility in *Listeria* requires **confirmation by MIC** and strong QC reporting; otherwise, the result risks being an artefact of interpretive criteria, media, inoculum, or reading.

**How to address** (minimum):

1.
  - Confirm vancomycin with MIC testing for at least a representative subset (ideally all isolates called resistant).
  - Report QC strains and acceptable ranges; describe plate media, disc potencies, incubation conditions (already partly provided) plus QC performance.
  - Avoid framing vancomycin resistance as a “severe threat” until confirmed by MIC.
2. **Species identification resolution**

- The study uses 16S rRNA gene sequencing and BLAST-based identification.

16S may not fully resolve closely related *Listeria* species/lineages.

**How to address**: Add a confirmatory approach (species-specific PCR targets, MLST/cgMLST, or WGS on a subset), or explicitly acknowledge identification limits and avoid overinterpreting “global clones/novel strains” claims.

**3) Are sufficient details of methods and analysis provided to allow replication by others?**

**Answer: Partly**

**What is adequately described**

- Sampling logistics and composite creation are described.
- Enumeration steps and plating strategy are described (duplicate plating, selective media, incubation).
- QMRA framework and parameter definitions are clearly presented (including conservative assumptions).

**Missing details needed for reproducibility**

- **AST raw data**: Provide inhibition zone diameters (mm) per isolate/antibiotic (or as a supplement), not only “R/S percentages.”
- **Quality controls**: Identify QC strains used for disk diffusion and PCR (positive/negative controls, extraction controls).
- **Isolate counts and denominators**: Clarify how many isolates were tested in AST vs how many positives in enumeration. The abstract-level statement refers to large numbers (e.g., “156 strains”), but the study also reports 80 positive samples; this needs transparent linkage.
- **Bioinformatics details**: For sequence analysis, specify trimming/assembly criteria, alignment length, and accession list mapping to isolate IDs and sample metadata (produce/region).

**4) If applicable, is the statistical analysis and its interpretation appropriate?**

**Answer: Partly**

**Strengths**

- The clustering and correlation visualization approach is described (Jaccard distance, average linkage).

**Concerns and recommendations**

- **Correlation matrix interpretation**: If Pearson correlations are presented, specify if variables are binary/ordinal and whether Pearson is appropriate; include p-values or confidence intervals, or frame as exploratory.
- **QMRA uncertainty**: The QMRA uses deterministic assumptions such as F_path=0.10, P=1 (100% prevalence), R=1 (no reduction), and standard serving size.

These assumptions are explicitly “worst case,” which is acceptable, but then the authors should:

-
  - include **sensitivity analysis** (which parameter drives risk most),
  - include **uncertainty analysis** (Monte Carlo or scenario analysis),
  - provide justification and local context for “frequency of consumption” and “serving size” choices.

**6) Are the conclusions drawn adequately supported by the results?**

**Answer: Partly**

**Supported elements**

- The study demonstrates widespread detection of *Listeria* spp. in sampled produce and provides an internally consistent QMRA framework based on measured loads.

**Overreach and how to address it**

- **Severity of threat vs modeled risk**: The authors note annual illness probabilities are very low (10⁻⁹–10⁻¹⁰) while also using strong language about immediate threat.

This should be balanced by clarifying population-level framing and model conservatism.

- **Mechanistic claims not directly supported**: The conclusion suggests vancomycin resistance is “probably” due to plasmid-based van genes transferred from Enterococci.

That may be plausible but is not demonstrated here (no plasmid analysis, no WGS context, no transfer experiments). This should be reframed as a hypothesis and supported with citations, or removed.

**Points that MUST be addressed to make the article scientifically sound**

1. **Confirm key AMR findings with appropriate methodology**, especially vancomycin: perform MIC testing (at least subset; ideally all “R” isolates) and report QC procedures and strains.
2. **Provide transparent breakpoint documentation** and reconsider non- *Listeria* breakpoint use (e.g., *Staphylococcus* breakpoints for oxacillin/cefoxitin). If retained, clearly label as exploratory and avoid clinical-resistance claims based on these breakpoints.
3. **Clarify the unit of analysis and limitations from composite sampling and** ensure prevalence/occurrence metrics are not overinterpreted as individual sample prevalence.
4. **Improve reproducibility by releasing underlying data** (enumeration, AST raw zones, gene detection calls, QMRA spreadsheet/code). Current “data availability” covers sequences only.
5. **Moderate claims of “novel strains/global clones”** unless additional typing/WGS is provided; otherwise reframe as “sequence diversity observed in 16S rRNA data.”

**Minor suggestions **

- Align discussion strength with QMRA outputs and clearly differentiate “worst-case scenario” assumptions (P=1, R=1) from typical consumer behaviors.
- Provide a clear flow diagram: samples → composites → positives → isolates characterized → isolates in AST/genotyping/QMRA.
- Add a short limitations paragraph summarizing: composite sampling, 16S resolution limits, breakpoint limitations, deterministic QMRA.

Is the work clearly and accurately presented and does it cite the current literature?

Partly

If applicable, is the statistical analysis and its interpretation appropriate?

Partly

Are all the source data underlying the results available to ensure full reproducibility?

Yes

Is the study design appropriate and is the work technically sound?

Partly

Are the conclusions drawn adequately supported by the results?

Partly

Are sufficient details of methods and analysis provided to allow replication by others?

Partly

Reviewer Expertise:

food microbiology

I confirm that I have read this submission and believe that I have an appropriate level of expertise to confirm that it is of an acceptable scientific standard, however I have significant reservations, as outlined above.

# Reviewer report: Reviewer response for version 1

**DOI:** 10.5256/f1000research.189332.r450239

**Published:** 2026-02-03

**Authors:** Rahul Vashishth

**Affiliations:** 1 Vellore Institute of Technology, Vellore, Tamil Nadu, India

The manuscript requires only minor corrections.  This is a well-designed and technically sound study, with methods explained clearly enough for others to replicate. The statistical analyses are appropriate, and the interpretations are logical.  Overall, the conclusions are well supported by the results, and the manuscript reads clearly. 1. Adding some recent studies from 2021–2026 could further strengthen the discussion and provide better context for the findings.

 2. Is the McFarland value stated in the manuscript accurate?

Is the work clearly and accurately presented and does it cite the current literature?

Partly

If applicable, is the statistical analysis and its interpretation appropriate?

Yes

Are all the source data underlying the results available to ensure full reproducibility?

Yes

Is the study design appropriate and is the work technically sound?

Yes

Are the conclusions drawn adequately supported by the results?

Yes

Are sufficient details of methods and analysis provided to allow replication by others?

Yes

Reviewer Expertise:

Microbilogy,Molecular Biology

I confirm that I have read this submission and believe that I have an appropriate level of expertise to confirm that it is of an acceptable scientific standard.

# Response

**Published:** 2026-02-03

**Authors:** Anthony Adegoke

The authors appreciate the reviewers for the constructive contribution. We also want to appreciate the observation as follows:

  1. Adding some recent studies from 2021–2026 could further strengthen the discussion and provide better context for the findings.

 Response: Once again, thank you. We will update this as suggested by the reviewer.

 2. Is the McFarland value stated in the manuscript accurate? Yes. 0.5 McFarland standard was intended. The approx. value is 1.5 x 10 ^8^ CFU/mL. We will use this value used this specific value in the research. We are grateful for this important point.
